# DNA Double Strand Break Repair Pathways in Response to Different Types of Ionizing Radiation

**DOI:** 10.3389/fgene.2021.738230

**Published:** 2021-09-30

**Authors:** Gerarda van de Kamp, Tim Heemskerk, Roland Kanaar, Jeroen Essers

**Affiliations:** ^1^Department of Molecular Genetics, Erasmus MC Cancer Institute, Erasmus University Medical Center, Rotterdam, Netherlands; ^2^Oncode Institute, Erasmus University Medical Center, Rotterdam, Netherlands; ^3^Department of Vascular Surgery, Erasmus University Medical Center, Rotterdam, Netherlands; ^4^Department of Radiation Oncology, Erasmus MC Cancer Institute, Erasmus University Medical Center, Rotterdam, Netherlands

**Keywords:** linear energy transfer, double strand break repair, combination therapy, DNA repair pathway, end resection

## Abstract

The superior dose distribution of particle radiation compared to photon radiation makes it a promising therapy for the treatment of tumors. However, the cellular responses to particle therapy and especially the DNA damage response (DDR) is not well characterized. Compared to photons, particles are thought to induce more closely spaced DNA lesions instead of isolated lesions. How this different spatial configuration of the DNA damage directs DNA repair pathway usage, is subject of current investigations. In this review, we describe recent insights into induction of DNA damage by particle radiation and how this shapes DNA end processing and subsequent DNA repair mechanisms. Additionally, we give an overview of promising DDR targets to improve particle therapy.

## Introduction

Over the past decades, the interest in particle radiotherapy for the treatment of tumors has been on the increase. This is illustrated by the fact that, as of 2021, about 100 proton therapy centers are operational world-wide (Paganetti et al., 2021). In addition, a few carbon ion irradiation centers have been established and are used for the treatment of patients (Tinganelli and Durante, 2020). The central rationale for particle therapy is its superior spatial dose distribution in tissue in comparison to conventional X-ray therapy. Photons deposit the maximum dose at the entrance of the tissue, followed by a gradually decline of dose throughout the remaining tissue. In contrast, protons and other particles deposit a relatively low dose at the entrance while at a certain depth the dose sharply increases, forming the so-called Bragg peak (Newhauser and Zhang, 2015). In this way the major dose is delivered to the tumor and the dose delivered to the surrounding tissue is minimized. The main advantage of this dose distribution is the sparing of so-called organs at risk, resulting in less irradiation-induced side-effects.

Both relative biological effectiveness (RBE) and linear energy transfer (LET) are used to describe differences between particle radiation and X-ray radiation. The RBE is defined as the ratio of the reference radiation type absorbed dose to the absorbed dose of a radiation type that induces the same biological endpoint, for example cell survival (Gulliford and Prise, 2019). X-rays with a defined energy or cobalt-60 γ-rays are often used as reference radiation type. The LET is defined as the amount of energy that a particle transfers to the material traversed per unit distance (Gulliford and Prise, 2019). Radiation types are usually divided into low LET radiation and high LET radiation. Examples of low LET radiation are X-rays or γ-rays and examples of high LET radiation are α-particles and carbon ions. Protons have a relatively low LET compared to α-particles and carbon ions. However, the LET varies throughout the Bragg curve and is, compared to photons, especially higher in the Bragg peak.

Currently, the RBE in clinical proton therapy is taken to be 1.1 (Paganetti et al., 2002; Paganetti, 2014). In comparison, the RBE of, for example, α- particles is significantly higher, with reported values ranging from 3 to 15 (Durante et al., 1995; Thomas et al., 2003; Franken et al., 2012). The use of a standard RBE of 1.1 in clinical proton therapy is based on measured RBE values *in vivo* from some of the very first proton studies (Dalrymple et al., 1966; Urano et al., 1980). However, there are several uncertainties regarding the RBE of the proton beam, since there are differences in LET throughout the Bragg curve. Especially, in the Bragg peak and at the distal edge of the Bragg peak the LET is significantly higher compared to photon radiation. Additionally, there is a substantial variability in results from both *in vitro* and *in vivo* studies studying the RBE of protons (Paganetti et al., 2002; Paganetti, 2014). This variability can be explained by the fact that the RBE is not only dependent on the LET, but also on other physical factors, such as energy and dose rate of the proton beam, and biological factors, such as type of tumor, cell cycle stage and oxygenation level (Vitti and Parsons, 2019; McNamara et al., 2020). To get insight in the effective RBE of protons and which factors determine the effective RBE, more studies directly comparing the proton versus the photon response in defined cell and *in vivo* models, using defined beam characteristics have to be performed (Durante et al., 2019). In addition, studying cellular responses after high LET irradiation in defined models gives insight into the effect of LET on the RBE and sheds light on the possible added value of high-LET irradiation therapy, such as carbon ion therapy, in comparison to proton therapy (Ma et al., 2015; Nagle et al., 2016; Tinganelli and Durante, 2020).

An important determinant of the effectiveness of radiotherapy is the repair of the DNA damage that is induced by the radiation. In particular, DNA double strand breaks (DSBs) are considered to be a determinant of cell survival since they can lead to cell death if left unrepaired. In this review we provide an update on recent insights into the repair of DNA damage induced by different radiation types. To this aim we provide an overview of the DNA damage response (DDR) upon ionizing radiation (IR), the determinants of the different options between DNA end protection and resection and how the arising substrate can undergo subsequent DNA repair. Additionally, we will give an overview of combination therapies that can potentially be implemented to exploit the properties of particle therapy.

## Induction of DNA Damage by Different Types of Radiation

Photon radiation induces mainly isolated lesions including single strand breaks (SSBs), base damage and DSBs. In contrast, particle radiation with high LET, such as α-particles and carbon ions are thought to induce a more highly localized and clustered DNA damage (CDD). The LET of protons varies throughout the Bragg curve and therefore the spatial distribution of the induced lesions by protons might be different throughout the Bragg curve. Usually, CDD is defined as two or more lesions formed within one or two helical turns of the DNA. However, this definition does not indicate anything about the type of lesions. For example, DNA damage clusters can consist of non-DSB lesions, such as SSBs and base damage or a DSB with nearby non-DSB lesions or a cluster of DSBs, containing multiple DSBs in close proximity (Nickoloff et al., 2020). It is important to have a clear definition and characterization of CDD, since different DNA lesions could have different effects on DNA repair mechanisms.

It is widely appreciated that the complexity and yield of radiation-induced CDD increases with increasing LET. However, this view might be oversimplified, since particles are physically different from each other and from photons in, for example, energy, charge and diameter (Jezkova et al., 2018). To get insight into the induction of DNA damage by different particles in comparison to photons, the spatial configurations of the induced DNA damage has to be determined. Monte Carlo simulations of the induction of clustered DNA lesions by IR are, at present, the only means to predict the spatial configurations of individual lesions per cluster (Georgakilas et al., 2013). Visualizing DNA damage clusters in cells by use of (immuno)fluorescence microscopy is challenging, since a high resolution is needed to separate individual lesions (Natale et al., 2017). Several studies have shown that the use of electron microscopy (EM) can overcome this resolution barrier (Lorat et al., 2015; Timm et al., 2018). However, EM has certain disadvantages compared to (immuno)fluorescence microscopy, such as the infeasibility to do live-cell imaging and the limited options for labeling DNA repair proteins, which hamper the systemic and thorough understanding of radiation-induced cellular responses.

One of the first events after induction of DSBs by IR is the phosphorylation of histone H2A.X, also referred to as γH2A.X, and the accumulation of the DDR protein 53BP1 which forms so-called IR induced foci (IRIF) at the site of the break (Panier and Boulton, 2014). Immunostaining and fluorescent microscopy imaging of these foci has revealed that γH2A.X and 53BP1 foci are larger after proton irradiation compared to photon irradiation (Szymonowicz et al., 2020). High-resolution stimulated emission depletion (STED) microscopy has shown that these larger foci consist of several individual sub-foci (Szymonowicz et al., 2020). This suggests that either the foci observed after proton irradiation consist of multiple lesions or that the chromatin condensation is different around the induced breaks (Lopez Perez et al., 2016; Natale et al., 2017). Additionally, foci induced by protons remain longer compared to those induced by photons, indicating that they are repaired less efficiently (Oeck et al., 2018; Szymonowicz et al., 2020). Similar observations have been made in cells irradiated with other particles, such as α-particles and carbon ions (Nagle et al., 2016; Roobol et al., 2020). 53BP1 foci induced by α-particle are bigger than foci induced by photons (Roobol et al., 2019). Following live dynamics of GFP-tagged 53BP1 foci in α-particle irradiated cells has shown that the repair of DSBs is slower after high LET IR compared to low LET IR. This indicates that these lesions are different from each other and are also repaired differently.

The production of closely spaced lesions rather than individual lesions by particle irradiation is considered crucial for mutagenesis, genomic instability and cell death (Georgakilas et al., 2013). However, how the type of DNA damage caused by different irradiation types correlates with DNA repair mechanisms and subsequent mutagenesis or cell death is not fully understood. Therefore, more studies characterizing the configurations of particle-induced DNA damage and studying subsequent DNA repair and cellular responses are needed.

## DNA Damage Response

The induction of DNA lesions by IR triggers a cascade of cellular responses, called the DDR, that includes localization and recognition of the lesions which ultimately leads to the repair of the induced damage. DNA damage often triggers cell cycle arrest, and when not properly repaired, apoptosis and cellular senescence. DSBs initiate a cell cycle arrest through checkpoints in G1 and G2 phase of the cell cycle (Shaltiel et al., 2015). These checkpoints prevent the replication and segregation of the damaged DNA, which is crucial for maintenance of genomic integrity. The induction of either cell cycle arrest, apoptosis or cellular senescence can be mediated by the transcriptional regulator p53 which is phosphorylated and activated by ATM. After induction of DSBs variations in p53 protein levels regulate the induction and duration of cell cycle arrest and apoptosis by controlling the expression of a wide variety of target genes (Loewer et al., 2013; Reyes et al., 2018).

Upon DSB induction, the Mre11/Nbs1/Rad50 (MRN) complex accumulates at the DNA damage, bridges the two DNA ends and activates ATM (Reginato and Cejka, 2020). One of the first events after the induction of a DSB is the phosphorylation of histone H2A.X on Serine 139 of its C-terminal tail by the DDR kinases ATM, ATR and DNA-PKcs up to megabases flanking the DNA damage site (Scully and Xie, 2013). MDC1 binds directly to γH2A.X and functions as a scaffolding protein that is thought to mediate most of γH2A.X functions (Stucki et al., 2005). However, MDC1 can also bind to chromatin in a γH2A.X independent manner, indicating that MDC1 might have additional γH2A.X-independent functions (Salguero et al., 2019). MDC1 mediates chromatin methylation and ubiquitination by functioning as a docking site for RNF8 which subsequently leads to the recruitment of RNF168. Ubiquitination of histones by RNF8 and RNF168 initiates a downstream cascade that is crucial for the localization of downstream DNA repair factors. Several proteins, which are involved in different DNA repair pathways, such as the BRCA1-BARD1 complex, 53BP1, and the MRN complex are localized to the site of DNA damage and mediate resection or protection of the DNA ends.

## End Processing

**FIGURE 1:**
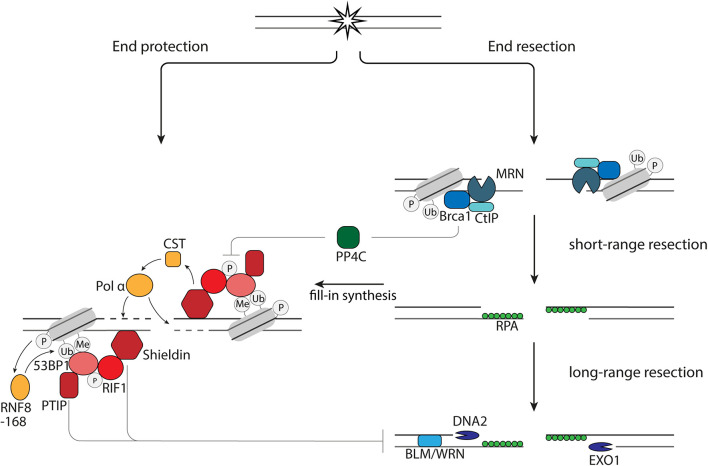
End resection versus end protection. 53BP1 protects DNA ends via its effector proteins, PTIP, RIF1, and Shieldin via (1) inhibition of EXO1 and DNA2 and (2) fill-in synthesis by the CST-Pol α. End protection is counteracted by BRCA1 via dephosphorylation of 53BP1 by PP4C.

DNA repair pathways that can act on DNA ends are homologous recombination (HR), single strand annealing (SSA), non-homologous end joining (NHEJ) and theta-mediated end joining (TMEJ). Which pathway is used for the repair of DSBs follows from the enzymes that act at the DNA end. The DNA repair pathways will be discussed in further detail in the next section of this review. This section will focus on processing of the DNA end and how this is influenced by the cell cycle stage, differentiation stage, and complexity of the DNA damage (Figure 1).

The first short-range end resection of DNA ends is performed by the endonuclease Mre11 in complex with Rad50 and Nbs1 (Figure 1). Subsequently, long range 5′-3′ end resection can occur, which is executed by the nucleases EXO1 and DNA2 in collaboration with BLM or WRN helicase (Figure 1). The single strand DNA (ssDNA) strands that arise during resection are bound by RPA to protect them from degradation and forming secondary structures. While the nucleases have a direct role in resecting the DNA ends, BRCA1 and 53BP1 have indirect roles in regulating resection. The unique interaction between 53BP1 and BRCA1 is illustrated by the fact that loss of 53BP1 can reverse lethality of BRCA1-deficient cells and mice (Bouwman et al., 2010). This rescue is, at least in part, mediated by restoration of HR through an increase in end resection due to the loss of 53BP1 and BRCA1-independent, RNF168-mediated localization of Rad51 (Bunting et al., 2010; Zong et al., 2019). Although it is widely appreciated that 53BP1 inhibits end resection in G1 phase of the cell cycle, it is also shown that 53BP1 in S and G2 phase of the cell cycle plays a role in inhibiting hyperresection, allowing limited resection and repair by HR rather than SSA (Ochs et al., 2016).

When 53BP1 binds to the site of DNA damage, it is phosphorylated by ATM. This phosphorylation leads to accumulation of PTIP and RIF1. Loss of RIF1 or its effector protein, Shieldin, are epistatic with 53BP1 deletion in sensitization to DSB-inducing therapies, increase of end resection after induction of DSBs and rescue of HR in BRCA1-deficient cells (Setiaputra and Durocher, 2019). The inhibition of resection by Shieldin is mediated by CST-Polα-mediated fill-in synthesis, since CST prevents end resection, interacts with Shieldin and accumulates at DNA damage sites in complex with pol α in a 53BP1- and Shieldin-dependent manner (Figure 1; Barazas et al., 2018; Mirman et al., 2018). However, this might not be the only way by which end resection and subsequent repair is influenced by the presence of Shieldin at DNA ends. Disruption of the 53BP1-PTIP interaction in BRCA1-deficient cells rescues end resection, but not Rad51 loading (Callen et al., 2020). Rad51 loading in these cells is restored when the Shieldin subunit, Shld3, is depleted (Callen et al., 2020). This study also shows that PTIP prevents long-range end resection by DNA2, while Shieldin prevents long-range end resection by EXO1 (Callen et al., 2020). In summary, this shows that the 53BP1 effector axes, RIF1-Shieldin and PTIP are both important for protection of DNA ends and might have differential effects on DNA repair.

Binding of BRCA1 to the DNA ends results in release of RIF1 from the site of DNA damage, as resection in BRCA1-deficient cells is rescued by RIF1 depletion. Upon irradiation there is an increased amount of RPA foci in these cells as the result of restored resection. Additional depletion of the phosphatase PP4C does not increase the amount of RPA foci, while RPA foci increase in cells containing 53BP1 phosphorylation mutants. This shows that the release of RIF1 is the result of dephosphorylation of 53BP1 by PP4C (Isono et al., 2017). Whether a similar mechanism is applicable for PTIP is not known. An important regulator of BRCA1 and thereby end resection is CtIP. CtIP can be post-translationally modified on different sites. Post-translational modifications of CtIP are important for the bridging of DNA ends, stimulation of Mre11 activity, interaction with BRCA1, localization of BLM and EXO1 at DNA ends and enhancement of DNA2-mediated long-range end resection (Wang et al., 2013; Anand et al., 2016; Daley et al., 2017; Ceppi et al., 2020; Öz et al., 2020).

One of the factors that influences the processing of DNA ends is the cell cycle stage. End processing in different cell cycle stages is controlled by a number of factors, including CDKs and cyclins (Hustedt and Durocher, 2017). The level of CDKs is low in G1 phase, but rises during S and G2 phases. In S/G2 phase, CDKs promote end resection by phosphorylating CtIP, EXO1, DNA2, and Nbs1 (Chen et al., 2011; Mirman et al., 2018; Öz et al., 2020). Additionally, factors involved in either end processing are differentially expressed in the different cell cycle stages. For example, the expression of some of the proteins involved in end resection, such as CtIP and Mre11, is higher in S and G2-phase than in G1-phase (Kanakkanthara et al., 2016). The chromatin surrounding the DSB is another cell cycle regulated factor that influences end resection. 53BP1 binding to γH2A.X domains is dependent on the additional chromatin marks H4K20me2 and H2AK15ub. The ubiquitination of H2A on lysine 15 is mediated by RNF168 and thus the direct result of DSB induction (Mattiroli et al., 2012). In contrast, H4K20me2 is present throughout the cell cycle. However, upon replication in S-phase this histone mark is diluted by incorporation of H4K20me0 histones, which allows accumulation of the BRCA1-BARD1 complex, displacement of 53BP1 and end resection (Simonetta et al., 2018; Nakamura et al., 2019).

Additionally, end processing is influenced by the differentiation stage of the cells. Embryonic stem (ES) cells proliferate fast and have a relative short G1 phase (Vitale et al., 2017). Despite their fast proliferation, ES cells repair DNA damage with high fidelity and have a low mutation accumulation rate. This high fidelity repair is mainly attributed to the fact that repair of DSBs in ES cells is less dependent on NHEJ compared to differentiated cells (Bañuelos et al., 2008; Tichy, Pillai et al., 2010). Higher expression levels of proteins that promote end resection such as BLM, WRN, and BRCA1 in ES cells in comparison to differentiated cells probably contribute to this phenomenon by enhancing end resection and directing the substrate into homology directed repair (Maynard et al., 2008). End protection of telomeres is mediated by the Shelterin complex. TRF2 is an essential protein of this complex and prevents the end-to-end fusion of telomeres by NHEJ. However, TRF2 is not essential for end protection of telomeres in ES cells (Ruis et al., 2021). This example illustrates that differentiated and non-differentiated cells might use different mechanisms to safeguard the integrity of their genome.

Another factor that influences the processing of DNA ends, is the complexity of the DNA damage. As described before particles are thought to induce more CDD compared to photons. Therefore, they can be used as a tool to study the effect of complexity of DNA damage on DNA repair. Specifics about the radiation sources used and biological models in the studies that are cited throughout this review can be found in Table 1. Several studies compared particle-induced and photon-induced end resection. For example, the percentage of RPA foci positive cells is increased in cells irradiated with high-LET particles compared to photon-irradiated cells (Averbeck et al., 2014)Additionally, iron and carbon ions induce more RPA and CtIP phosphorylation compared to γ- and X-rays (Yajima et al., 2013). Moreover, CtIP depletion impairs repair of carbon ion induced DNA damage, but not of X-ray induced DNA damage, indicating that resection is an essential step in the repair of carbon ion induced lesions (Yajima et al., 2013; Averbeck et al., 2014). A recent study shows that α-particle-induced foci contain multiple RPA foci (Roobol et al., 2020). These findings suggest that DNA ends of DNA damage induced by high LET radiation are more prone to end processing compared to DNA ends of DNA damage induced by low LET radiation.

**TABLE 1:** Overview of studies investigating DNA repair after particle irradiation.

**Process**	**Study model**	**Radiaton type**	**LET (keV/μm)**	**Energy**	**Read out**	**References**
End resection	U2OS (siRNA Ctip, Mre11, and Exo1)	X-ray	2	250 keV	Clonogenic survival	Averbeck et al., 2014
	AG1522D	12C ions	90	100 MeV/nucleon	RPA foci	
	MEFs (H2AX−/−, Ku80−/−)		170	11.4 MeV/nucleon	γH2A.X foci	
	NFFhTERT (siRNA Ctip)	40Ca ions	200	186 MeV/nucleon		
		59Ni ions	350	265 MeV/nucleon		
		14N ions	400	11.4 MeV/nucleon		
		48Ti ions	2180	11.4 MeV/nucleon		
		59Ni ions	3430	11.4 MeV/nucleon		
		119Sn ions	7880	11.4 MeV/nucleon		
		197Au ions	13000	11.4 MeV/nucleon		
		207Pb ions	13500	11.4 MeV/nucleon		
		238U ions	15000	11.4 MeV/nucleon		
End resection	U2OS	X-rays			RPA tracks/foci	Yajima et al., 2013
	HeLa	γ-rays			γH2A.X tracks/foci	
	U251	12C ions	70	290 MeV/nucleon	Western blot (p-RPA, p-ATM,	
	MEFs	Fe ions	200	500 MeV/nucleon	p-Chk1, γH2A.X)	
	1BR-hTERT					
	48BR					
End resection	U2OS	X-ray		195 kV	53BP1 foci	Roobol et al., 2020
		Alpha particles	115 ± 10	5.486 MeV	RPA foci	
	MEF LIG4−/−	X-ray			Clonogenic survival	
	MEF RAD54−/−	Proton	Entrance plateau		γH2A.X foci	
	MEF LIG4−/− RAD54−/−		protons			
NHEJ and HR	BxPC3 (BRCA2-proficient)		Center SOBP	100-109.9 MeV		Szymonowicz et al., 2020
	Capan-1 (BRCA2-deficient)					
	M059J (DNA-PKcs −/−)					
	M059K (DNA-PKcs+/+)					
NHEJ and HR	M059J (DNA-PKcs −/−)	X-ray		6 MeV	Clonogenic survival	Bright et al., 2019
	M059K (DNA-PKcs+/+)	Proton	1.1, 2.5, and 7.3	100 MeV	γH2A.X foci	
	HCC1937 (BRCA1 −/−)					
	HCC1937-BRCA1 (BRCA1 complemented)					
	HT1080 (wt, shRad51, shDNA-PKcs)					
NHEJ and HR	A549	X-ray		200 keV	Clonogenic survival	Fontana et al., 2015
	A549-DNA-Pkcs inhibitor NU7026	Proton	Center SOBP	138 MeV	γH2A.X foci	
	A549-siRNA DNA-PKcs					
	A549-siRNA Rad51					
	M059J (DNA-PKcs −/−)					
	M059K (DNA-PKcs+/+)					
NHEJ and HR	AA8 (WT and siRad51)	X-ray		200 keV	Clonogenic survival	Grosse et al., 2014
	Irs1sf (HR deficient XRCC3 −/−)	Proton	Center SOBP	138 MeV	γH2A.X foci	
	CH09 (WT)				Chromosomal abberations	
	UV5 (ERCC5 −/−)					
	XR-C1 (DNA-PKcs−/−)					
NHEJ and HR	H1299	X-ray		150/200 keV	Clonogenic survival	Ma et al., 2015
	H1299 + DNA PK inhibitor NU7026	12C ions	50, Center SOBP	290 MeV/nucleon	γH2A.X flow cytometry	
	H1299 + Rad51 inhibitor B02					
NHEJ and HR	3D PDAC tumors + inhibitors (B02, NU7026)	X-ray		200 keV	3D tumoroid formation	Görte et al., 2020
	BxPC3	Proton	3.7, Center SOBP	150 MeV		
	MiaPaCa2					
	Panc-1					
	Patu8902					
	Capan-1					
	COLO357					
NHEJ and HR	AA8 (WT)	γ-rays (137 Cs)		662 keV	Clonogenic survival	Carter et al., 2018
	V79 (WT)	Proton	2.2, Center SOBP	200 MeV	γH2A.X foci	
	Irs1sf (HR deficient XRCC3 −/−)	12C ions	50, Center SOBP	290 MeV/nucleon	Chromosomal abberations	
	Irs1 (HR deficient XRCC2 −/−)					
	XR1 (XRCC4 −/−)					
	V3 (DNA-PKcs −/−)					

*This table provides an overview of the radiation parameters and biological models used in the cited studies: the specific DNA repair process studied, cell model used (including information about protein knock-out or knockdown), radiation type, LET, energy of the particles, and the read-out that was used to study the indicated process.*

## Double Strand Break Repair Pathways Contribution Is Influenced by Resection Range

**FIGURE 2:**
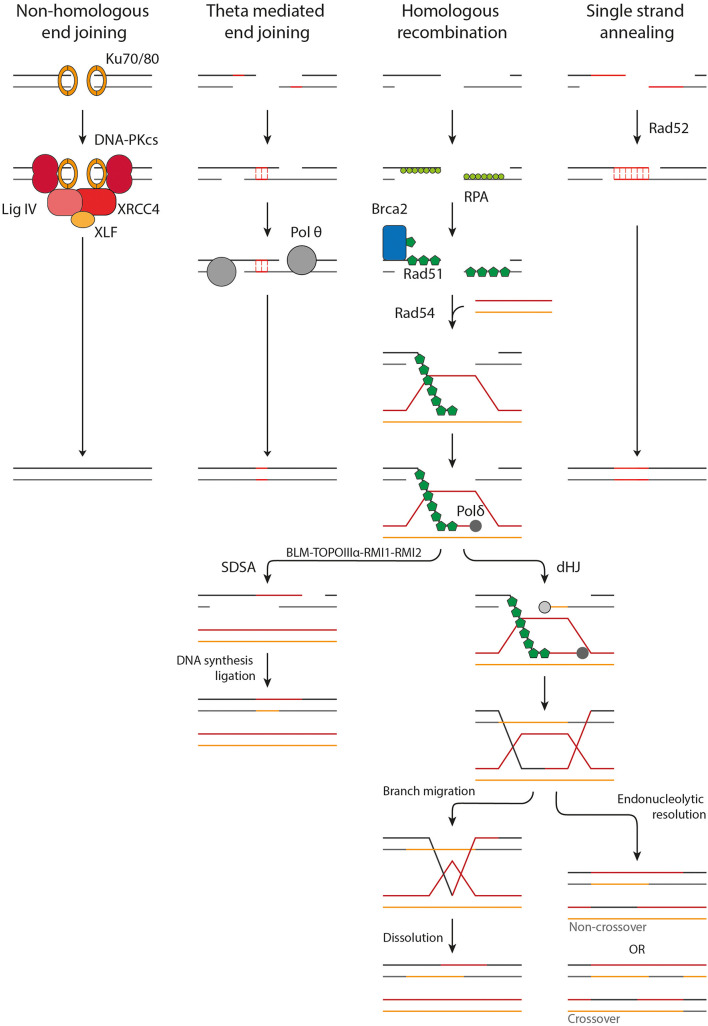
Overview of the DSB repair pathways. NHEJ acts upon protected DSB ends. Ku70/80 binds to the DSB ends followed by the accumulation of DNA-PKcs, XRCC4, XLF, and LigIV. The DSB ends are processed and ligated. TMEJ acts on short-range resected ends. Microhomology of 2–20 bp is required to transiently align the DSB ends, after which the gaps are filled in by Pol θ. HR requires long-range resection and makes use of a homologous donor sequence for repair. After resection Rad51-loading onto the ssDNA is mediated by BRCA2. Homology search is facilitated by Rad54. Once a homologous donor strand has been found a D-loop is formed and DNA synthesis is started. In SDSA the D-loop is disrupted, followed by DNA synthesis and ligation to repair the DSB. A D-loop can also progress into the formation of a dHJ, which can be resolved by branch migration and dissolution or endonucleolytic resolution. Endonucleolytic resolution can result in a non-crossover or crossover product.

In this section the DNA repair mechanisms NHEJ, HR, SSA and TMEJ and their activity upon (particle) radiation-induced DSBs will be discussed (as illustrated in Figure 2).

The first major pathway of DSB repair following X-ray irradiation is NHEJ. NHEJ acts mainly on DSB ends protected from resection by 53BP1, as described above. First, the DSB end is bound by the Ku70/80 heterodimer. Ku70/80 forms a ring structure which interacts with the sugar phosphate backbone in a sequence-independent manner (Walker et al., 2001). It has a high affinity for dsDNA ends, including blunt ends and 5′ and 3′ overhangs, but has a significantly lower affinity for long stretches of ssDNA (Mimori and Hardin, 1986; Ono et al., 1994; Fell and Schild-Poulter, 2015). DNA-PKcs locates to Ku70/80 and orchestrates the repair by phosphorylating other downstream factors, such as Artemis and XRCC4. DSB ends produced by IR are often not directly ligatable due to mismatching overhangs, damaged nucleotides or bulky adducts, but need processing first (Liao et al., 2016; Pannunzio et al., 2018). This task is mainly performed by Artemis, a versatile endonuclease, and three DNA polymerases, namely pol μ, pol λ and terminal deoxynucleotidyl transferase (TdT). After processing, the DSB ends can be ligated by the DNA ligase IV/XRCC4 dimer, which is enhanced by XLF and PAXX (Wyman and Kanaar, 2006; Pannunzio et al., 2018; Zhao B. et al., 2020). In contradiction to its name, NHEJ can make use of limited sequence homology (<4 bp) between the overhangs of the DSB ends, however, this does not require end resection. While NHEJ does usually not restore the original sequence, it does restore the structural integrity of the DNA quickly. Hereby it prevents inappropriate end joining and translocations that could lead to loss of genetic material, chromosome aberrations, and carcinogenesis (Zhao B. et al., 2020; Zhao L. et al., 2020).

A second major pathway for DSB repair is HR. In contrast to NHEJ, HR acts upon long-range resected ends and uses more than 100 bp of homology. HR is initiated by loading of Rad51 onto the ssDNA (Wyman and Kanaar, 2006; Wright et al., 2018). Rad51 forms helical filaments on ssDNA that acts as a scaffold for itself and for other interacting proteins. Rad51 filament formation is hindered by RPA which is bound to the ssDNA. BRCA2 mediates the loading of Rad51 by displacing RPA and acting as nucleation platform for Rad51 (Wright et al., 2018). BRCA2 sequestration of Rad51 is suggested to prevent inappropriate Rad51-DNA interactions (Reuter et al., 2014). The Rad51 filament is not a static structure but changes filamental pitch based on ATP hydrolysis. These local changes in filament pitch promote nucleoprotein filament movement (Wright et al., 2018). The formation of the nucleoprotein filament, also called the presynaptic complex (PSC), potentiates recognition of a homologous donor. Homology recognition and strand invasion are mediated by binding of Rad54 to Rad51 (Crickard et al., 2020). This interaction is dependent on bromodomain containing protein, BRD9 (Zhou et al., 2020). Upon induction of DNA damage, Rad54 is acetylated on Lysine 515 (K515-Ac). BRD9 binds K515-Ac on Rad54 and facilitates Rad54’s interaction with Rad51, which is essential for HR (Zhou et al., 2020). Recent single-molecule studies have shown that Rad54 changes the homology search from a diffusion based search to an ATP dependent motor-driven mechanism. The current hypothesis is that Rad54 reinforces the binding of the PSC to a dsDNA donor, after which it can be scanned thoroughly for homology. Upon binding of the PSC to a donor strand, the dsDNA is transiently separated to allow Rad51 to probe for homologous sequences. This transient melting of the DNA is most likely mediated by RPA and the Rad54 motor activity, influencing the DNA topology. It was also shown that both donor DNA strands can be sampled for homology in the presence of RPA, revealing a new role for RPA in homology search (Crickard et al., 2020).

Once a complementary sequence has been located, the synaptic complex is formed. The 3′ end of the invading strand can intertwine with the donor DNA, forming heteroduplex DNA (hDNA) suitable for DNA synthesis. The formed structure is called a D-loop. Rad54 motor activity again plays an important role in this process, likely by altering the topological state of the DNA (Wright et al., 2018). This hDNA structure is bound by DNA polymerase, mainly polymerase δ, after which DNA synthesis can commence. The D-loop can be processed by two pathways, synthesis-dependent strand annealing (SDSA) or by formation of a double Holliday junction (dHJ). In somatic cells, a D-loop is more likely to be processed by SDSA then by dHJ formation. This probably has evolved because dHJs can result in crossover products, which can lead to loss of heterozygosity of critical genes such as tumor suppressors (Wyman and Kanaar, 2006; Wright et al., 2018).

In SDSA, DNA synthesis extends the invading strand such that it has sufficient overlap with the other DSB end once the D-loop is disrupted. After disruption of the D-loop by the BLM-TOPOIIIα-RMI1-RMI2 complex, the ssDNA ends can anneal to each other and DNA synthesis can commence from the other DSB end by a still unidentified polymerase. After a final ligation of the remaining nicks, the DSB is repaired and the DNA is restored. In dHJ formation the remaining 3′ ssDNA end not associated with the homologous donor also invades the D-loop. DNA synthesis occurs on both donor strands followed by ligation of the nicks, resulting in the formation of a dHJ. These HJs can be processed by either dissolution or endonucleolytic resolution. In dissolution, the HJs are brought together via branch migration (Wright et al., 2018). Recently, it has been shown that branch migration is mediated by the N-terminal domain of Rad54 (Goyal et al., 2018). When the two HJs meet they can be resolved, leading strictly to non-crossover. With endonucleolytic resolution the phosphodiester backbone is cleaved across the HJ. If the cleavage is in the same plane it results in non-crossover but if the HJs are cleaved in different planes a crossover product is formed (Wyman and Kanaar, 2006; Wright et al., 2018). HR is usually error-free because it makes use of a sister chromatid as a template for repair. This dependence on a template limits its activity to late S and G2 phase (Wyman and Kanaar, 2006; Wright et al., 2018).

A third pathway that acts on resected DNA ends is TMEJ. This pathway is considered as an alternative end joining pathway, since it only makes use of microhomologies of 2-20 bp. Because of this, TMEJ is often referred to as alternative end joining (alt-EJ) or microhomology-mediated end joining (MMEJ). Here, we will use the term TMEJ to distinguish between the theta-mediated end joining pathway and alternative end joining in general, which may encompass other end joining activity. If a homologous sequence is present, TMEJ can act upon ssDNA revealed by short range end-resection (∼20 bp) (Zhao B. et al., 2020; Xue and Greene, 2021). Whether TMEJ can also act upon long-range resected ends remains to be proven (Truong et al., 2013). During TMEJ the DSB ends are transiently aligned using the revealed microhomologies. An important protein in this process is polymerase θ (pol θ). Pol θ is an A-family DNA polymerase with helicase activity. It can displace RPA from ssDNA and it plays an important role in searching and aligning the microhomologies (Mateos-Gomez et al., 2017; Sallmyr and Tomkinson, 2018). The non-homologous 3′ tails that remain after annealing of the microhomologies are removed, presumably by the structure specific nuclease complex XPF/ERCC1. Subsequently, the remaining gaps can be filled in by pol θ (Seol et al., 2018). Finally, ligation is performed by DNA ligase IIIα-XRCC1 (Sallmyr and Tomkinson, 2018). TMEJ is intrinsically mutagenic due to the deletion of a copy of the microhomology the sequence in between the microhomology sites.

The fourth form of DSB repair is SSA, which is also considered an alternative repair pathway and is less frequently activated. SSA acts upon long-range resected DNA ends (>1000 bp), utilizing homologies of more than 50 bp to anneal homologous sequences (Zhao B. et al., 2020; Xue and Greene, 2021). The key player in SSA is Rad52 which interacts with the RPA coated ssDNA and aligns the complementary regions. The 3′ tails are cleaved off by XPF/ERCC1, after which the gaps can be filled in by an unidentified polymerase. The final ligation is performed by DNA ligase I (Sallmyr and Tomkinson, 2018; Zhao B. et al., 2020). SSA is intrinsically mutagenic and is associated with larger deletions due to the deletion of one of the copies of the annealed repeat and the large sequence in between the complementary sites (Zhao B. et al., 2020).

The used DSB repair pathway to repair radiation-induced DSBs is not a choice as such, but rather it is dictated by the amount of resection, as well as the available resources, such as microhomologies, sister chromatids and repair proteins. Although the DSB repair pathways are often described conceptually as isolated pathways, flexible, and reversible interactions between the various DSB repair factors occur, eventually leading to repair of the DSB. As described in the previous sections, high LET particle-induced damage is thought to have a differential configuration than X-ray induced damage, usually termed CDD. By which pathway this different type of DNA damage is repaired, is still subject of current investigation. However, some studies studying DNA repair in cells depleted of key DNA repair proteins by CRISPR/Cas9 or siRNAs have been performed. Generally, NHEJ deficiency sensitizes to all types of radiation (Grosse et al., 2014; Fontana et al., 2015; Gerelchuluun et al., 2015; Ma et al., 2015; Bright et al., 2019; Görte et al., 2020), although the effect is less pronounced with high LET radiation (Ma et al., 2015; Bright et al., 2019). Various studies show that DSBs induced by low LET protons are mostly repaired by HR while DSBs induced by X-ray are predominately repaired by NHEJ. However, others do not observe this radiosensitization by HR knockdown for low LET protons (Gerelchuluun et al., 2015; Görte et al., 2020) or it is only observed for high LET protons (Bright et al., 2019) or carbon ion radiation (Gerelchuluun et al., 2015; Ma et al., 2015) (see Table 1 for details on the experimental setup). Unfortunately, these studies are difficult to compare due to differences in radiation energy, type, dose and the used biological model.

There are several possible explanations for why particle-induced DNA damage is more likely to be repaired by HR rather than NHEJ. High LET proton irradiation induces CDD consisting of DSBs, SSBs and abasic sites, while this is not the case for X-ray or low LET proton irradiation (Carter et al., 2018). Long ssDNA tails or ssDNA gaps near the DSB end can block Ku70/80 binding, hereby channeling the DSB into resection (Zhao L. et al., 2020). There might also be steric hindrance at the site of the CDD with multiple repair proteins competing to repair the different types of lesions. Resection is not hampered by the presence of abasic sites or SSBs, hereby making resection more favorable. Another possibility is that with higher LET more lesions are created, leading to exhaustion of the DNA repair protein pool, preventing end-protection (Roobol et al., 2020). 53BP1 can protect DSB ends from resection up to 20–40 simultaneous DSBs. If the DSB load exceeds this maximum capacity, the 53PB1 pool is exhausted, leading to resection and RPA loading (Ochs et al., 2016; Roobol et al., 2020). Interestingly, 53BP1 exhaustion, i.e., all available 53BP1 in the nucleus is chromatin bound, does not lead to upregulation of HR. At high doses, Rad51 focus formation is decreased and recombination efficiency is reduced (Ochs et al., 2016; Mladenov et al., 2020). This effect is not induced due to exhaustion of the Rad51 pool since only 20% of available Rad51 is chromatin bound at maximum level of foci observed (Mladenov et al., 2020). Instead, high doses of IR induce hyperresection of breaks, which promotes SSA (Ochs et al., 2016; Mladenov et al., 2020). It is hypothesized that 53BP1 does not prevent resection entirely but instead fosters HR rather than the mutagenic SSA pathway (Ochs et al., 2016). Interestingly, evidence also exist that suggests that Rad51 focus formation and, by inference, HR is upregulated after knock-out of 53BP1 (Mladenov et al., 2020). Additional research studying repair pathway choice and the underlying mechanisms after induction of DSBs by particle-radiation is needed to fully unravel the differential contribution of the different repair pathways. This knowledge will provide rationales for combining particle radiation therapies with DDR targeting therapies.

## Combination Therapies

**FIGURE 3:**
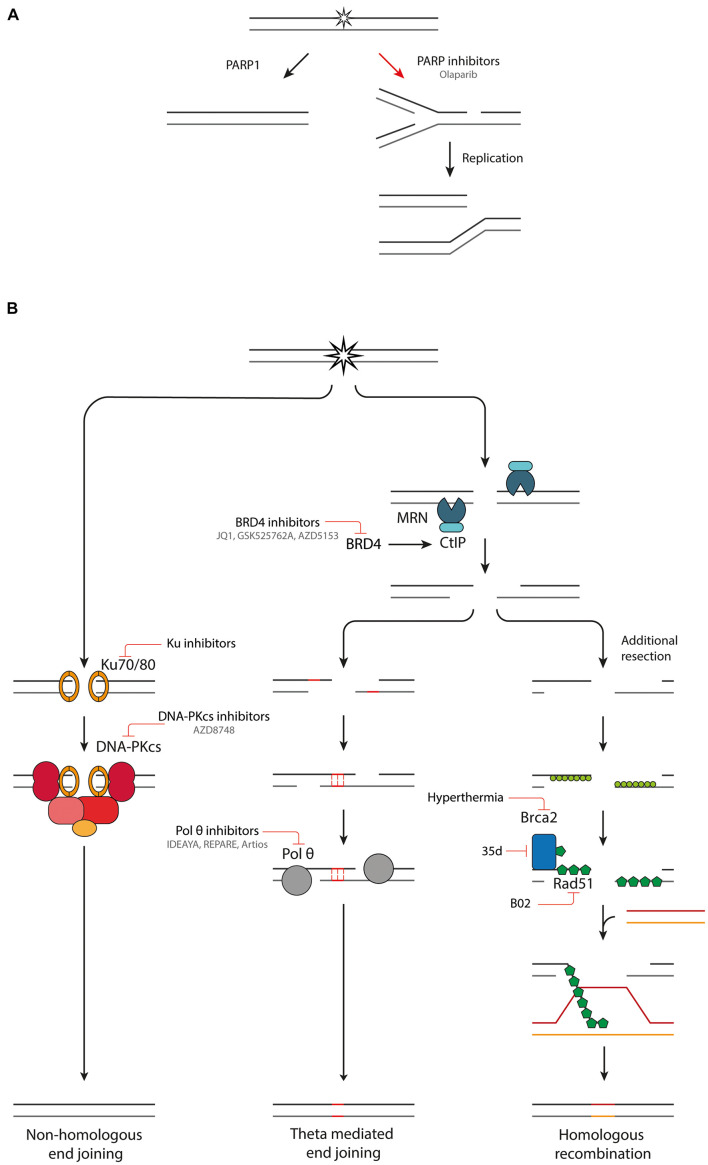
Combination therapies targeting DDR pathways. **(A)** Inhibition of PARP1 leads to the conversion of SSBs into DSBs upon DNA replication. **(B)** An overview of NHEJ, TMEJ and HR and inhibitors targeting various components of these pathways.

Particle radiotherapy is a promising treatment modality for the treatment of cancer, especially due to their superior spatial dose distribution in comparison to conventional X-ray therapy. However, the efficacy of particle radiotherapy could be further increased by combining it with inhibitors of DDR pathways. A potential strategy would be to exploit the difference in induced damage by the low LET entrance dose in healthy tissue, and the higher LET Bragg peak in the tumor. Another promising strategy is the induction of synthetic lethality, whereby a genetic defect in a DDR pathway is exploited using pharmacological inhibitors of compensatory DDR pathways. This can lead to cell death and genomic instability in the tumor, while the healthy tissue is spared, since it does not carry this DDR mutations (Pilié et al., 2019; Reuvers et al., 2020). This has sparked a great interest in the development of small molecule inhibitors of components of DDR pathways (Figure 3).

### Poly (Adp-Ribose) Polymerase Inhibition

The poly (ADP-ribose) polymerase (PARP) family comprises a group of DDR proteins that mainly function to detect SSBs and DSBs, localize DNA repair proteins and stabilize replication forks during DNA repair (Pilié et al., 2019). Synthetic lethality can be achieved with PARP inhibitors (Figure 3A). Upon inhibition of PARP1, repair of SSBs is attenuated, and unrepaired breaks can be converted to one-ended DSBs during replication. This leads to replication fork collapse which requires HR for repair (Bryant et al., 2005; Wyman and Kanaar, 2006; Nonnekens et al., 2016). Tumors which are HR deficient (HRD), such as with BRCA1/2 mutations, cannot efficiently repair this damage, leading to an anti-tumor effect (Farmer et al., 2005; Pilié et al., 2019). Multiple PARP inhibitors, such as Olaparib, have been developed, some of which have reached clinical trials and FDA approval for treatment of cancers with germline BRCA1/2 mutations (Pilié et al., 2019). PARP inhibitors can also be used in combination with radiotherapy. Next to inducing DSBs, radiation also induces other types of DNA damage such as SSBs. By PARP1 inhibition these SSBs are converted to DSBs, increasing the DSB burden after irradiation (Nonnekens et al., 2016). Hereby, the efficacy of low LET radiation is increased. High-LET radiation induces CDD of which a large part is SSBs (Carter et al., 2018). PARP inhibition could effectively transform high-LET induced CDD with SSBs and DSBs to a very complex DSB cluster, which in turn could increase the effectiveness of particle therapy. It was recently shown that Olaparib decreases survival of HeLa cells after relatively high LET proton irradiation, which is due to a deficiency in CDD repair. PARP inhibition had no impact on cell survival after low LET proton irradiation (Carter et al., 2019).

### Non-Homologous End Joining Inhibition

Non-homologous end joining reduces the efficacy of cancer treatment modalities such as radiation therapy, which rely on introducing DSBs. Inhibition of NHEJ greatly sensitizes tumor cells to radiotherapy. The radiosensitization effect is seen with multiple radiation modalities such as X-ray, proton and carbon-ion irradiation, across various cell lines, and in 3D tumor models (Grosse et al., 2014; Fontana et al., 2015; Gerelchuluun et al., 2015; Ma et al., 2015; Bright et al., 2019; Görte et al., 2020). The strongest radiosensitization is observed for low LET radiation (see Table 1 for details on the experimental setup) (Fontana et al., 2015; Gerelchuluun et al., 2015; Ma et al., 2015; Bright et al., 2019).

Small molecule inhibitors have been developed that target the first step in the NHEJ pathway, namely binding of Ku70/80 dimer to DNA (Figure 3B; Weterings et al., 2016; Gavande et al., 2020). The most promising inhibitors target a ligand binding pocket in close proximity to the DNA-binding region, interfacing with both the Ku70 and Ku80 subunit. By blocking the DNA binding capacity of the Ku heterodimer, the downstream catalytic activity of DNA-PKcs is inhibited, hereby preventing DSB repair (Weterings et al., 2016; Gavande et al., 2020). The inhibitors induce increased sensitivity to DSB inducing agents, such as IR, in *in vitro* experiments. Further validation of the specificity and potency in *in vivo* experiments is necessary before the Ku inhibitors can progress into clinical studies.

The next protein in the NHEJ pathway, DNA-PKcs, has had great research interest as a target for inhibition of NHEJ. However, it has been challenging to develop a DNA-PKcs inhibitor that selectively inhibits DNA-PKcs without affecting the structurally related PI3 lipid and PI3K protein kinase (Goldberg et al., 2020). Recently, a new DNA-PK inhibitor, AZD8748, was identified, which shows only weak activity against PI3K lipid kinases and no significant off-target effects. Furthermore, AZD7648 is a potent DNA-PKcs inhibitor and an efficient sensitizer to radiation- and doxorubicin-induced DNA damage in models of non-small-cell lung cancer (NSCLC) cells and xenografts, as well as patient derived xenograft models. These promising results have led to the progression of AZD7648 to clinical studies (NCT03907969) (Fok et al., 2019; Goldberg et al., 2020).

Artemis is an important nuclease in NHEJ, responsible for processing the DSB ends. Without it, processing of ‘dirty’ DSBs, which are commonly produced by radiation, is hampered (Kanaar et al., 2008; Zhao B. et al., 2020). Artemis inhibition could influence break structure and thus affect which downstream enzymes can further act on the break. Structure-based research into small molecule inhibitors have been hampered by a lack of crystal structure. However, the recent publication of the crystal structure of the catalytic domain of Artemis opens up new opportunities for structure-based design of selective Artemis inhibitors (Karim et al., 2020).

### Polymerase θ Inhibition

Inhibition of pol θ is another promising avenue for induction of synthetic lethality. Cells with defective HR machinery are reliant on TMEJ for DSB repair (Feng et al., 2019; Kamp et al., 2020; Schrempf et al., 2021). Using a CRISPR genetic screen, Pol θ inhibition is synthetically lethal with many proteins important in replication associated DSB repair (Feng et al., 2019). Currently there are two described mechanisms for the synthetic lethality between pol θ and HR inhibition. First, TMEJ is important for the repair of one-ended DSBs from collapsed replication forks, which would normally be repaired by HR. In the absence of effective HR machinery, cells become reliant on TMEJ to repair these lesions. This idea is supported by the fact that TMEJ inhibition synergizes with PARP inhibitors (Schrempf et al., 2021). The second mechanism is the anti-recombinase activity of pol θ. Pol θ contains Rad51 binding motifs and antagonizes Rad51-mediated recombinase. When the HR machinery is defective, pol θ is necessary to remove the Rad51 and to allow repair by other means. There is evidence that without pol θ there is accumulation of toxic Rad51 complexes preventing further repair (Ceccaldi et al., 2015; Cleary et al., 2020; Schrempf et al., 2021). TMEJ is especially interesting as target since it is unlikely to have a large effect on the survival of healthy tissues proficient in NHEJ and HR. However, a sensitization will be observed in cancer cells with defective DDR machinery, which are more reliant on TMEJ for repair of DSBs (Sallmyr and Tomkinson, 2018). Pol θ inhibitors are being developed by three independent biotech companies: IDEAYA, REPARE therapeutics and Artios Pharma (Figure 3B). The first clinical trials with pol θ inhibitors are expected to already start in 2021 (Schrempf et al., 2021).

### Homologous Recombination Inhibition

As mentioned above, PARP inhibitor treatment can greatly increase tumor response in HRD tumors. The last two decades, work has been focused at expanding the utility of PARP inhibitor treatment, by looking into the possibility of inducing a HRD state in HR proficient tumors, to induce synthetic lethality. This has led to the development of small molecule inhibitors for various HR proteins (Figure 3B; Carvalho and Kanaar, 2014). Next to their utility in combination with PARP inhibition, HR small molecule inhibitors can also sensitize tumor cells to radiation. The radiosensitization of HR inhibition has been studied in the context of low and high LET irradiation (see Table 1 for details on the experimental setup) (Grosse et al., 2014; Fontana et al., 2015; Gerelchuluun et al., 2015; Ma et al., 2015; Bright et al., 2019; Görte et al., 2020). So far, the results have been contradictory on whether HR inhibition sensitizes cells to proton irradiation. HR inhibition-induced radiosensitization has been observed with low LET protons (Grosse et al., 2014; Fontana et al., 2015). However, others only observe the radiosensitization effect for high LET protons (Bright et al., 2019) or only with carbon ions with an even higher LET (Gerelchuluun et al., 2015; Ma et al., 2015). A common trend in these studies is that HR inhibition radiosensitization increases with LET. This could make HR inhibitor treatment especially promising in combination with particle therapy. When administering a DDR inhibitor systemically, both healthy, and tumor tissue will be affected. Combined with radiotherapy this could lead to severe side effects. Although there are some contradicting results, evidence shows that HR inhibition radiosensitizes cells predominantly to high LET radiation, only present in the Bragg peak targeted at the tumor, and not to low LET radiation which hits the surrounding healthy tissue. Hereby toxicity to healthy tissue could be reduced, increasing the therapeutic window.

Several pharmaceutical inhibitors of HR are currently under development. A promising target for inducing HRD is bromodomain containing 4 (BRD4) inhibition. BRD4 is a member of the bromodomain and extraterminal (BET) protein family and facilitates oncogene transcription. Multiple small molecule inhibitors can selectively target BRD4 such as JQ1, GSK525762A and AZD5153. There are multiple ongoing clinical trials with BRD4 inhibitors or more general BET inhibitors (NCT01587703 and NCT03059147) (Sun et al., 2018). The mechanism by which BRD4 inhibition treatment induces HRD has been resolved recently. Next to variably affecting expression of many DDR proteins, a consistent downregulation of CtIP is observed with four different BRD4 inhibitors. CtIP interacts with the MRN complex, promoting end resection of DSB breaks and inducing nuclease activity of the MRN complex. Downregulation of CtIP impairs HR. Furthermore, BRD4i works synergistically with PARP inhibition, hereby inducing synthetic lethality (Sun et al., 2018).

Small molecule inhibitors that directly interfere with Rad51 have also been developed. A particularly interesting target is the disruption of Rad51-BRCA2 binding. BRCA2 is an important mediator in Rad51 loading onto ssDNA (Wright et al., 2018). Without this interaction Rad51 loading is greatly reduced. A new series dihydroquinolone pyrazoline derivatives have been designed that target the LDFE binding pocket of Rad51 (Bagnolini et al., 2020). The compound 35d inhibits the protein-protein interaction between Rad51 and BRCA2, by binding to Rad51 and is capable of reducing HR efficiency. Furthermore, in combination with PARP inhibition it induces synthetic lethality in pancreatic cancer cells. Unfortunately, its low solubility currently prevents it from further studies in *in vivo* models.

B02 is a small molecule inhibitor that interferes with the DNA binding capacity of Rad51, hereby inhibiting DNA strand exchange and branch migration (Huang et al., 2012). B02 sensitizes breast cancer cells to various types of chemotherapy *in vitro* and in a xenograft model (Huang and Mazin, 2014). In combination with radiotherapy B02 shows a radiosensitizing effect to photon and proton irradiation in NSCLC and pancreatic cancer cells. This effect was even further increased in combination with PARP inhibition treatment (Wéra et al., 2019).

Apart from chemical inhibition of repair proteins, physical procedures can also influence pathway effectivity such as temperature and oxygenation status (Krawczyk et al., 2011; Luoto et al., 2013).

Chronic tumor hypoxia downregulates expression of key HR proteins (Chan et al., 2009). Rad51 is downregulated by hypoxia in multiple tumors by transcriptional repression (Bindra et al., 2004). BRCA1 expression is also downregulated in hypoxic cells which could alter the resection of DSBs and shunt them into NHEJ (Bindra et al., 2005). Hypoxia-mediated downregulation of RNA expression is not only observed for HR genes (e.g., Rad51, Rad52, Rad54, BRCA1, and BRCA2), but some NHEJ genes are also affected (e.g., Ku70, DNA-PKcs, DNA Ligase IV, and XRCC4). However, this downregulation of NHEJ-related RNA expression does not appear to result in an altered protein level (Meng et al., 2005). This hypoxia-mediated downregulation of HR is also reflected in a decreased recombination efficiency and increased sensitivity to DNA cross-linking agents (Chan et al., 2008). The above mentioned studies were performed under moderate (0.1–0.5%) to severe (<0.1%) tumor hypoxia, however, under mild hypoxia (0.5–2.5%) these effects might be less pronounced or absent. Furthermore, the duration of hypoxia influences the effects as well since these effects are only observed under chronic hypoxia (>48 h) and not under acute hypoxia.

Tumor hypoxia hampers effective radiotherapy by an increased radioresistance of hypoxic cells, due to a decreased level of free oxygen radicals during irradiation (Bindra et al., 2004). The use of high LET particle radiation is promising for the eradication of hypoxic cells. The oxygen enhancement ratio (OER), defined as the ratio of doses given under hypoxic and normoxic conditions to produce the same biological effect, decreases with increasing LET. However, the benefit of using carbon-ions instead of protons was shown to be relatively moderate (1–15%) at clinically relevant oxygen levels (Wenzl and Wilkens, 2011). However, by exploiting the HRD in the hypoxic cell population, novel therapies could be used to selectively target these cells.

Hyperthermia is considered to be one of the most potent radiosensitizers. During hyperthermia treatment, the tumor region is heated locally to temperatures in the range of 40–44°C, using specialized equipment (Horsman and Overgaard, 2007; Van Den Tempel et al., 2017). Hyperthermic radiosensitization can be attributed to many macroscopic and microscopic biological effects in the tumor such as improved tumor oxygenation and DDR modulation (Van Den Tempel et al., 2017; Elming et al., 2019). One of the more recently described effects is hyperthermia-induced HRD (Krawczyk et al., 2011; Van Den Tempel et al., 2017). Upon subjecting cells to hyperthermia, BRCA2 is degraded, hereby inhibiting Rad51 loading onto resected 3′ends and preventing HR. It has been established that optimal HR inhibition is reached by subjecting cells to hyperthermia at 41-43°C for 30 to 60 min (Van Den Tempel et al., 2017), and that BRCA2 degradation is mediated by the proteasome (Krawczyk et al., 2011; Van Den Tempel et al., 2019). In both cultured cells and fresh patient material, Rad51 focus formation is abolished after hyperthermia application (Krawczyk et al., 2011). Because of the reduced HR, tumor cell are dependent on other, more error-prone, and DSB repair pathways. This results in a higher number of translocations after irradiation (Bergs et al., 2013). Furthermore, a synergistic effect is reached by combining PARP inhibitor treatment with hyperthermia (Krawczyk et al., 2011). With the help of hyperthermia, a HRD status can be induced in innately HR proficient tumor cells, hereby inducing synthetic lethality. The main advantage of hyperthermia over small molecule inhibitors is the targeting possibility of hyperthermia. By locally applying hyperthermia, HRD is only induced in the tumor region, hereby preventing systemic effects (Krawczyk et al., 2011; Van Den Tempel et al., 2019).

## Concluding Remarks

There is rising interest in particle radiotherapy for the treatment of tumors. This is mainly based on its superior dose distribution in comparison to photons. However, there is insufficient understanding on how cells and tumors engage the DDR in response to particle irradiation, which is a crucial process in determining the effectiveness of the therapy. DNA damage induced by high LET radiationis currently collectively referred to as CDD which reflects the fact that high LET radiation induces different types of DNA lesions compared to photons and that different particles have different lesion spectra. More research using markers for DSBs and other types of lesions will improve the understanding of this CDD. Furthermore, mechanistic understanding of whether and how this damage induces an altered DDR is still lacking. Studies comparing photon and particle induced DNA repair will shed light on the differential DNA repair mechanisms of particle-induced DNA damage and photon-induced DNA damage. Moreover, these studies will reveal fundamental molecular knowledge about factors that can be involved in differential end resection or protection and subsequent DNA repair pathways. This knowledge is crucial for further improvement of radiotherapy, since it opens up new possibilities for the rational design of combination therapies with DDR inhibitors that could potentially further increase the efficacy and applicability of radiotherapy.

## Author Contributions

GK, TH, and JE: conceptualization. GK, TH, JE, and RK: writing, reviewing, and editing. All authors contributed to the article and approved the submitted version.

## Conflict of Interest

The authors declare that the research was conducted in the absence of any commercial or financial relationships that could be construed as a potential conflict of interest.

## Publisher’s Note

All claims expressed in this article are solely those of the authors and do not necessarily represent those of their affiliated organizations, or those of the publisher, the editors and the reviewers. Any product that may be evaluated in this article, or claim that may be made by its manufacturer, is not guaranteed or endorsed by the publisher.
